# Current Landscape of Children's Surgery in Africa: A Multicenter Analysis of 16,000 Cases

**DOI:** 10.1002/wjs.70066

**Published:** 2025-09-05

**Authors:** Phillip J. Hsu, Madeleine Carroll, Alan Zambeli‐Ljepovic, Bolusefe Tijesuni Olatunji‐Banire, Pawan Mathew, Jason Axt, Thierno Diallo, Mekonen Eshete, Bertille Ki, Joseph Macharia, George Ngock, Absalat Serawit, Emma Bryce, Doruk Ozgediz, Maija Cheung

**Affiliations:** ^1^ Department of Surgery University of Michigan Ann Arbor Michigan USA; ^2^ Department of Surgery Yale University New Haven Connecticut USA; ^3^ Department of Surgery University of California San Francisco San Francisco California USA; ^4^ Kids Operating Room Edinburgh Scotland; ^5^ Department of Surgery Kijabe Hospital Kijabe Kenya; ^6^ Department of Surgery Hôpital Général Idrissa Pouye Dakar Senegal; ^7^ Surgical Department School of Medicine Addis Ababa University College of Health Sciences Addis Ababa Ethiopia; ^8^ Yekatit 12 Hospital Medical College Plastic and Reconstructive Surgery Department Addis Ababa Ethiopia; ^9^ CHU Pediatrique Charles de Gaulle Ouagadougou Burkina Faso; ^10^ Department of Surgery Meru Teaching and Referral Hospital Meru Kenya; ^11^ Department of Surgery Mbingo Hospital Baingo Cameroon

**Keywords:** epidemiology, LMIC, outcomes, pediatric surgery

## Abstract

**Background:**

Although prior studies have estimated the burden of pediatric surgical disease in low‐ and middle‐income countries (LMICs) through statistical modeling and hospital‐ or household‐based surveys, few large‐scale descriptions of procedures and outcomes have been published. We aimed to describe the epidemiology and outcomes of children's surgical care at multiple centers across Africa.

**Methods:**

Perioperative clinical data were collected prospectively from 2018 to 2023 at 17 hospitals in 11 African countries using a preexisting tool. Data came from children (age < 18 years) who underwent a surgical procedure in facilities equipped by the NGO Kids Operating Room. Data were stored on REDCap and descriptively analyzed.

**Results:**

16,454 procedures were performed, with a higher frequency of procedures performed in younger children than in older children (mean age 4.5 years). Congenital malformations, acquired genitourinary conditions, and acquired gastrointestinal conditions made up the most common diagnoses. We found a mortality rate of 3.7%, with higher mortality in neonates compared to younger children; conditions associated with the greatest mortality included congenital conditions, intestinal perforation, burns, and intussusception. Emergent operations were associated with much higher rates of mortality than elective operations.

**Conclusions:**

For the first time at this scale, we have assessed the epidemiology and outcomes of pediatric surgical care in LMICs. Findings were consistent with studies on the burden of disease, with a larger proportion of younger children accessing surgery, comparable mortality to other African studies, and higher mortality than in HICs. Future research and multilevel advocacy are needed to identify gaps in care and to design more effective interventions to reduce global disparities in access to surgical care for children.

## Graphical abstract

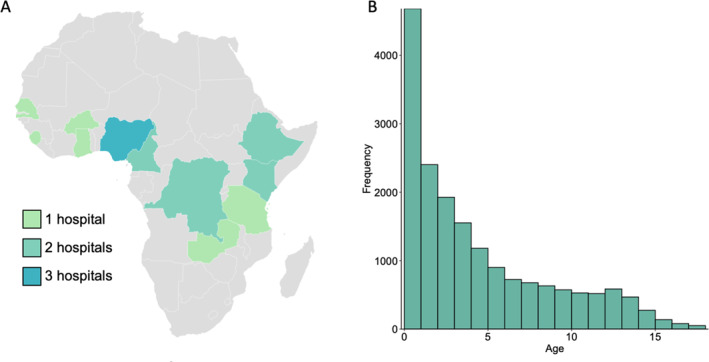

## 1 Introduction

Children constitute the majority of the population in low‐ and middle‐income countries (LMICs), yet they disproportionately bear the burden of untreated pediatric surgical disease. Although under‐5 mortality from communicable diseases decreased from 1990 to 2016, mortality from noncommunicable diseases, including surgical diseases, increased. Congenital anomalies rose from the seventh leading cause of childhood death to the fifth, ahead of HIV, TB, and malaria [1]. Furthermore, 90% of congenital anomalies are diagnosed in LMICs and up to two‐thirds are curable with cost‐effective surgical interventions [2, 3]. Surgical management of certain congenital anomalies and other pediatric surgical conditions is more cost‐effective in terms of cost per DALY averted than treatments such as antiretroviral therapy for HIV/AIDS(3). Yet, operative mortality rates for children in LMICs often exceed those in high‐income countries (HICs) by over 50% [4, 5]. Contributing challenges include inadequately trained staff, failure to implement safe surgery protocols, and insufficient facilities and equipment [6].

The existing lack of large‐scale data on conditions and outcomes in pediatric surgery in LMICs limits targeted interventions [7]. Most LMIC data come from household surveys or hospital‐based studies, which indicate that 6%–17.6% of children have an unmet surgical need [8, 9, 10, 11]. In Uganda, surgically treatable disease causes one‐third of all childhood deaths, and an estimated 7.2% of children had an untreated surgical condition, equating to 1,300,000 children nationwide [10]. Surveys in Nepal and Rwanda found similar figures, with an unmet pediatric surgical need of 6% and 6.3%, respectively [8, 9]. Given challenges in accurate data collection, inherent limitations of household‐based surveys, and unknown numbers of children dying before diagnosis, these figures likely underestimate the true burden.

We aimed to better understand the profile and burden of surgical diseases and outcomes in children that do access care. We analyzed cases performed in operating rooms (ORs) installed by Kids Operating Room (KidsOR) to compile the largest multi‐institutional study of pediatric surgical patients in Africa to date.

## 2 Methods

KidsOR is a philanthropic NGO dedicated to building pediatric operating rooms in LMIC hospitals. The organization employs a four‐pronged strategy: equipment and infrastructure, pediatric staff training, research capacity expansion, and advocacy for safe pediatric surgery [12]. The overall goal is to improve access to timely and safe children's surgery.

Perioperative clinical data were collected prospectively between 2018 and 2023 at 17 hospitals in 11 African countries, as a part of a collaboration between the hospitals and KidsOR, as previously described [13, 14] All available postinstallation data from hospitals with a KidsOR operating room were included. These sites were selected in collaboration with local governments, ministries of health, hospitals, and surgical teams to identify facilities where improving pediatric surgical infrastructure would be impactful. Selection factors included disease burden, existing healthcare capacity, and the commitment of local partners to maintain and staff the operating rooms.

Data came from all patients (age < 18 years) who underwent a surgery in a KidsOR operating room. A consensus‐driven data collection protocol developed by KidsOR and partners was used [13]. Prior to data collection, informed consent was obtained from each patient's caregivers. Data collection included two sources: caregiver interviews and patient medical records. Caregiver interviews provided insight into the patient's medical history, socioeconomic background, and experiences related to the surgery. Patient medical records were reviewed to gather clinical data, including diagnosis, surgical interventions, and postoperative outcomes.

Data were stored in a secure database hosted on REDCap. Mortality was defined as death prior to discharge. Analyses and statistical testing via chi‐squared tests were performed on deidentified data using RStudio V1.1.414. The study was approved by the Institutional Review Board of the University of California, San Francisco (#19‐29663) as well as by ethical review committees at each participating hospital.

This multicenter study involved the partner hospitals listed in Table 1, most of which are urban tertiary hospitals. Fifteen hospitals have fellowship trained general pediatric surgeons. Seven hospitals have trained pediatric anesthesiologists. The operating rooms at the remaining two hospitals focus on pediatric plastic reconstruction, with support from the SmileTrain organization.

**TABLE 1:** Hospital characteristics and number of patients.

Name	Location	Type	Pediatric surgeon present	Pediatric anesthesia present	*n*
CHU Pediatrique Charles de Gaulle, Burkina Faso	Urban	Tertiary	Yes	No	6141
Mbingo Baptist Hospital, Cameroon	Rural	Tertiary	Yes	Yes	43
Hôpital Gyneco‐Obstétrique et Pédiatrique de Yaoundé, Cameroon	Urban	Tertiary	Yes	No	106
Hospital Provincial General Reference De Bukavu, DRC	Urban	Tertiary	Yes	No	283
Centre Hospitalier Bethesda, DRC	Urban	Private referral	No	No	275
Menelik II Referral Hospital, Ethiopia	Urban	Tertiary	Yes	No	899
Yekatit‐12 Hospital, Ethiopia	Urban	Tertiary	Yes	Yes	672
Komfo Anokye Teaching Hospital, Ghana	Urban	Tertiary	Yes	No	649
AIC Kijabe Hospital, Kenya	Rural	Tertiary	Yes	Yes	851
Meru Teaching And Referral Hospital, Kenya	Urban	Tertiary, regional	No	Yes	100
Armed Forces Specialist Hospital, Nigeria	Urban	Military referral	Yes	Yes	225
Lagos University Teaching Hospital, Nigeria	Urban	Tertiary	Yes	Yes	504
National Hospital Abuja, Nigeria	Urban	Tertiary, national	Yes	No	1578
Hôpital Général Idrissa Pouye, Senegal	Urban	National	Yes	Yes	631
Connaught Hospital, Sierra Leone	Urban	Tertiary	Yes	No	373
Bugando Medical Centre, Tanzania	Urban	Tertiary	Yes	No	915
University Teaching Hospital (Lusaka), Zambia	Urban	Tertiary	Yes	No	2209
Total					16,454

## 3 Results

### 3.1 Demographics

**FIGURE 1:**
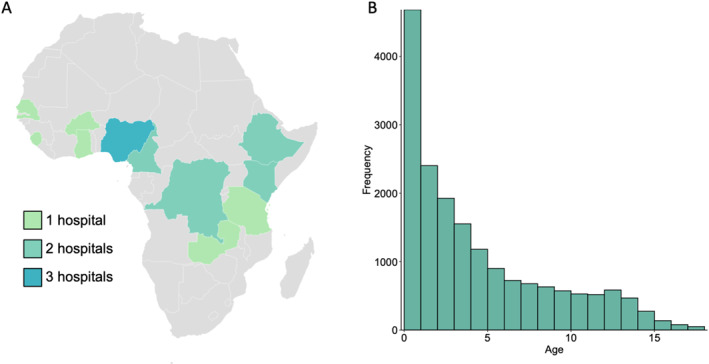
Distribution of (A) hospitals participating in this study and (B) patient age across all sites.

In total, 16,454 surgical procedures were performed on children at the 17 study hospitals (Figure 1A, Tables 1 and 2). Thirty‐three percent were female; when excluding diagnoses found exclusively or predominantly in males, such as inguinal hernias and genitourinary conditions, 43% were female. Mean age was 4.5 ± 4.4 years, and median age was 3 years, demonstrating a rightward skew. Concordantly, there was an increased proportion of younger patients compared to older patients (Figure 1B).

**TABLE 2:** Patient characteristics.

Age (years)	
Mean ± SD	4.5 ± 4.4
Median	3.0
Distribution (%)	
< 28 days	8.8
28 days to 1 year	16.5
1–5 years	38.2
5–12 years	26.0
12–18 years	10.4
Sex (female)	33%

### 3.2 Categories of Diagnoses and Procedures

**FIGURE 2:**
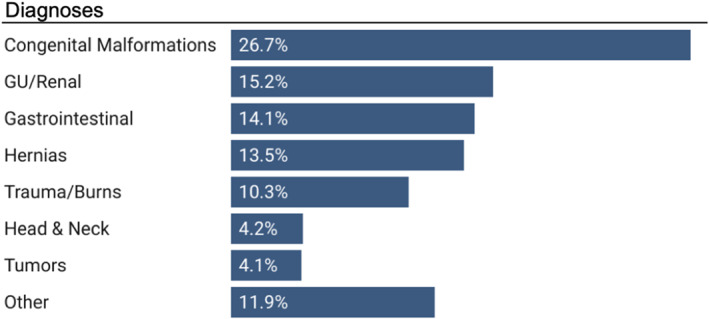
Frequency of diagnoses grouped by category.

The most common diagnosis categories were congenital malformations (*n* = 4390, 27%), acquired genitourinary/renal conditions (*n* = 2502, 15%), and acquired gastrointestinal conditions (*n* = 2502, 15%) (Figure 2, Table S1). Inguinal hernia was the most common individual diagnosis (*n* = 1413, 8.6%), followed by cleft lip/palate (*n* = 1264, 7.7%) and anorectal malformation (*n* = 924, 5.6%).

**FIGURE 3:**
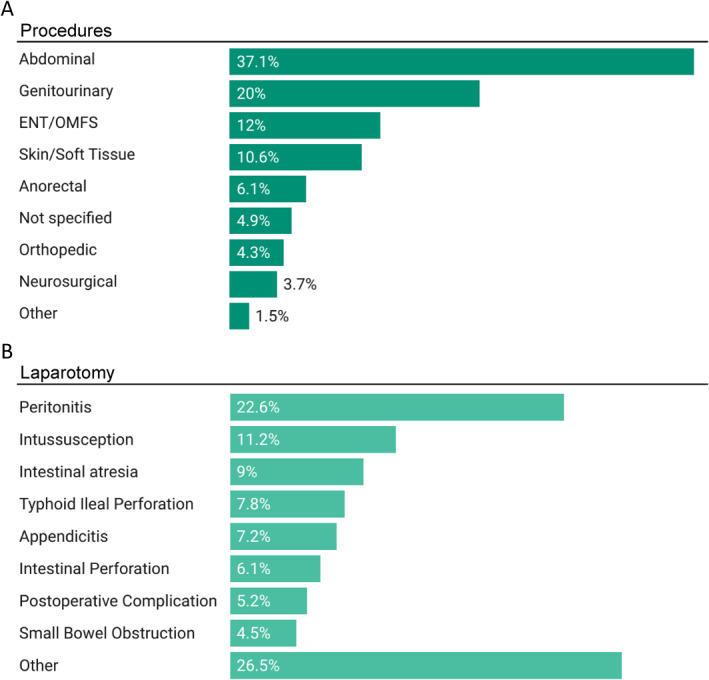
Frequency of (A) pediatric surgical procedures grouped by organ system and (B) diagnoses requiring laparotomy.

The most common procedure categories were abdominal (*n* = 6112, 37%), genitourinary/renal (*n* = 3283, 20%), and otolaryngology/maxillofacial (*n* = 1943, 12%) (Figure 3A). Laparotomy was the most common procedure overall (*n* = 1452, 8.8%) and was most often performed for primary peritonitis of unspecified etiology, intussusception, and intestinal atresia (Figure 3B). The next most common procedures overall were inguinal hernia repairs (*n* = 1413, 8.6%), cleft lip/palate repairs (*n* = 1229, 7.4%), ventral hernia repairs (*n* = 782, 4.8%), and circumcisions (*n* = 622, 3.8%).

### 3.3 Mortality

**FIGURE 4:**
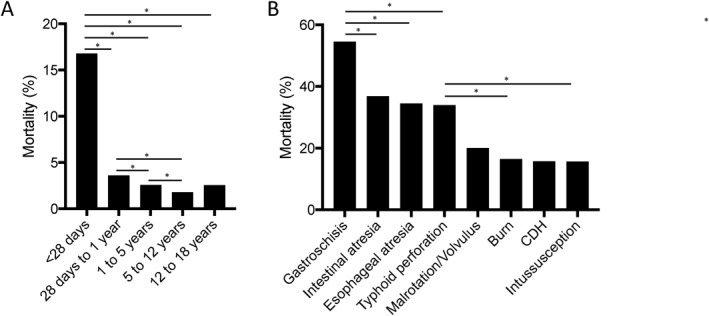
Frequency of mortality based on (A) age and (B) diagnosis. *Ddenotes *p* < 0.05.

The overall mortality rate was 3.7% (*n* = 580), of which 6% (33/580) occurred intraoperatively. Mortality was much higher in patients under 28 days of age (16.8%) compared to the other age groups (*p* < 0.001). Mortality was slightly higher in patients between 28 days and 1 year of age (3.6%) compared to patients between 1 and 5 years (2.6%, *p* = 0.013) and 5–12 years of age (1.8%, *p* > 0.001) (Figure 4A). Diagnoses with significant mortality included congenital conditions, intestinal perforation, burns, and intussusception (Figure 4B). Nearly 40% of deaths were associated with laparotomies, which had a mortality rate of 17.4% (*n* = 227/1304).

### 3.4 Emergency Surgeries

**FIGURE 5:**
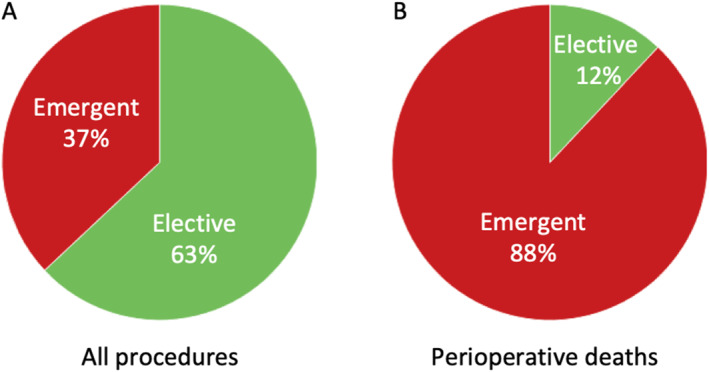
Frequency of (A) emergent to elective procedures and (B) mortality due to emergent and elective procedures.

Thirty‐seven percent (*n* = 6113) of all procedures were emergent. The conditions most commonly requiring emergency surgery were trauma/burns (*n* = 1194/6113, 20%), appendicitis (*n* = 451/6113, 7.3%), and inguinal hernias (*n* = 399/6113, 6.5%). In concordance, the most common emergent procedures performed were laparotomy (*n* = 1237/6113, 20%), wound closure (*n* = 400/6113, 6.5%), inguinal hernia repair (*n* = 372/6113, 6.1%), and appendectomy (*n* = 356/6113, 5.8%). The overall mortality rate for emergent procedures (9.1%, *n* = 512/6113) significantly higher than the mortality rate for elective procedures (0.7%, 68/9846) (*p* < 0.001) (Figure 5).

## 4 Discussion

We profiled the most common children's surgical diagnoses, procedures, and outcomes in 17 hospitals across 11 African countries. To our knowledge, this is the largest published multicenter study of pediatric surgical procedures in Africa. The treated disease burden we identified helps inform priorities in the future of children's surgery.

Prior epidemiological studies on pediatric surgical needs in LMICs found median ages of children requiring surgery to be 8–8.3 years, with 62.5% of children having at least one unmet surgical need [15, 16]. Our study extends this work by examining the patients accessing surgery. Neonates and infants comprised a substantial proportion of the patient population, contributing to a median age of 3 years, less than the mean age of 4.5 years. Congenital malformations constituted the most prevalent diagnosis category. Additionally, mortality was much higher in younger children compared with older children. This has several important implications.

Children require access to trained perioperative staff, including surgical, anesthesia, and nursing providers. Expertise in managing congenital conditions at all stages/ages is critical. Recent studies emphasize the broad spectrum of children's surgical needs and paradoxical lack of investment despite the cost‐effectiveness of interventions such as operating room installation [17]. The recently passed child health resolution, and WHO emergency, operative, and critical care resolutions, call for universal access to safe pediatric anesthesia and surgery [18, 19, 20]. Consensus guidelines (ORECS) provide a roadmap for gap analysis and implementation [18].

Because studied sites are largely urban, tertiary sites, the preponderance of neonates and infants in our cohort likely reflects case complexity and the recommendation that general anesthesia in children < 1 year be performed at tertiary centers. Additionally, older children with trauma and emergency surgical needs may be receiving treatment at district and regional hospitals. Even so, the volume of neonatal surgical need remains underreported due to death before surgery is accessed. In accordance with prior studies, our on‐site collaborators have reported long patient waiting lists due to high referral volume and limited pediatric surgical and perioperative workforce [13, 21].

Access is limited not only by lack of specialists but also by financial barriers causing delays in seeking, reaching, and receiving care [22, 23, 24, 25]. Traveling to hospitals equipped for pediatric surgery is a financial burden for many patients who must travel long distances [22, 23, 26]. Then, there is the cost of care, which is variable and often out‐of‐pocket [27, 28, 29]. The Africa Surgical Initiative 2030 identified that 70% of 32 surveyed African countries had no universal health insurance system [30]. Stigma presents an additional barrier to care, especially for congenital and oncologic pathologies with strong associated cultural beliefs. These factors underscore the importance of addressing systemic healthcare infrastructure, including establishing referral networks and improving rural access to comprehensive specialized care.

Recent work calls for policy implementation pursuing universal access to children's surgery. The Dakar Declaration emphasized that 1 in 3 African countries lack children's referral hospitals and most lack dedicated pediatric operating rooms or intensive care units. Their resolution called for integrating children's surgery into national health policy, including establishing at least one referral hospital for children per country [30]. The Pan‐African Surgical Healthcare Forum, which brought together over 30 African Ministries of Health, similarly compiled a consensus statement regarding the importance of surgical care plans, phased subnational implementation, and community level advocacy, while acknowledging the high proportion of children within the affected population [31].

Within the pediatric population, neonates are particularly vulnerable. We identified a neonatal perioperative mortality rate of 16.8%, compared to 0.3%–2.4% in HICs, emphasizing the size of existing gaps in prenatal and neonatal care [32]. These results build upon the landmark ASOS‐Paeds study, which found an in‐hospital mortality rate of 2.3% in children—11 times higher than in HICs [7]. A birth defect registry system, which is currently lacking in many African countries, would help inform priorities and resource allocation. Additionally, we must establish referral pathways involving general practitioners at rural district hospitals, community health workers, and home midwives. Such programs have been implemented in some countries, with ongoing expansion initiatives [33]. These are essential to reducing the neonatal surgical disease burden and improving outcomes for congenital and other disorders. For many conditions, such as intestinal atresias and abdominal wall defects, timely recognition—either prenatally or just after birth—is essential to prevent potentially lethal sequelae such as bowel perforation, respiratory distress, and dehydration. For neonates undergoing surgery, expert perioperative care is equally vital. Well‐equipped neonatal intensive care units with trained staff are needed for preoperative and postoperative resuscitation. However, safe perioperative care protocol implementation varies widely, and anesthesia and skilled nursing workforces are limited [34]. Intervention opportunities include utilizing published World Federation of Societies of Anesthesiologists and Global Initiative for Children's Surgery standards, new short educational courses developed in LMICs, simulation training, and continued large‐scale prospective data collection [35].

Another critical area of focus is specialized equipment for children's surgery. Many district hospitals, where most children first seek care, lack resources including water and electricity (unavailable in 25%), dedicated pediatric operating rooms (70.1%), blood transfusion within 2 h (73.7%), pediatric intensive care units (79.2%), and internet (96%) [30]. Our data came exclusively from Kids OR operating rooms equipped with specialized anesthesia machines, patient warmers, and surgical instruments. Despite this, mortality remained higher than in HICs, particularly in the youngest patients. Mortality rates at hospitals lacking specialized equipment are likely even higher. Although specialized facilities and equipment allow increased case complexity, and may thus increase mortality, previous studies showed no mortality increase mortality associated with higher operative volume after pediatric OR installation in Nigeria and Burkina Faso [14, 36].

Our study identified a high overall mortality rate of 3.7% and a very high emergency surgery mortality rate of 9.1%—13 times higher than for nonemergent cases. Outside congenital malformations, conditions with the highest associated mortality were typhoid perforations, burns, and intussusception, with rates similar to those in reported literature [37, 38, 39, 40, 41]. Among these conditions, the mortality rate of intussusception (15.6%) bore the most striking difference compared to that in HICs where mortality is < 1%. This difference is likely due to a number of factors, and serves as a representative example. It highlights the need for equipment and training to facilitate earlier diagnosis (i.e., ultrasound) and safe nonoperative management (i.e., pneumatic/hydrostatic reduction). Neither technique is widely practiced in Africa, though certain centers have demonstrated approaches that accommodate budgetary and workforce limitations [42]. However, clinical and epidemiologic context must be considered. Barriers to seeking care lead to delayed presentation, necessitating laparotomy regardless of the available resources. Addressing the high mortality of pediatric surgical emergencies will require a holistic approach that considers complex barriers at all phases of care.

An additional finding to address is our study population's unequal sex distribution, with a 2:1 male to female ratio. As we did not track data on patients who presented to a study hospital but did not undergo surgery, or those in the community with surgical conditions who never presented to a study hospital, we do not know to what degree this sex disparity is a reflection of unequal distribution of surgical disease burden versus differences in care‐seeking patterns. We believe it is likely a combination. Conditions, such as inguinal hernias and hydroceles, more common in or exclusive to males were among the most commonly treated surgical conditions. However, even excluding inguinal hernias and genitourinary/renal conditions, females make up only 43% of our population, suggesting other contributing factors. Prior studies found various sex distributions among pediatric surgical patients. Although some showed 1:1 ratios, others found similarly unequal ratios, with one from Connaught Hospital in Sierra Leone reporting a 2.6:1 male to female ratio [43]. Additionally, prior studies of care‐seeking behavior across many African countries have found that parents were more likely to seek care for male children [44].

Our study had several limitations. First, our database includes information from various Kids OR‐partnered hospitals, which differ in geographic distribution, accessibility, service availability (e.g., neurosurgery), and percentage of the pediatric surgical population served. As many of the KidsORs focus on general pediatric surgery, data from other specialties, such as neurosurgery or orthopedics, may be limited. Therefore, it cannot be assumed that our patient population is representative of the pediatric surgical population in any one country or in Africa overall. Second, only patients undergoing surgical procedures at said hospitals were included, excluding those not accessing care for surgical problems and those accessing care without surgery. Third, as data were largely deidentified, records were linked to procedures rather than patients, meaning patients with multiple procedures may be overrepresented. Fourth, our data collectors had a wide range of medical and research experience, with some having no prior training or experience in these areas and others being trained pediatric surgeons, which likely led to inconsistencies in coding. Fifth, we did not collect data on children who were operated on in adult ORs at participating hospitals, potentially contributing to the skewed age distribution and decreased representation of trauma and obstetric procedures. For example, at some study sites, emergent pediatric cases performed at night or on the weekends were done in other ORs. Variability in case mix/risk, disease burden, infrastructure, and surgical/perioperative expertise can create challenges in standardizing data collection, outcome measures, and comparisons across sites. The diversity of procedures—ranging from emergency trauma surgeries to elective pediatric interventions—makes it difficult to establish uniform inclusion criteria and outcome benchmarks. These factors can introduce heterogeneity that may limit the generalizability of findings and necessitate careful methodological design to account for contextual differences while maintaining study rigor. Furthermore, since these data reflect surgical practice after the installation of new operating room equipment, some centers may have undertaken more complex cases depending on their local priorities. Some sites were developed in partnership with SmileTrain, with an emphasis on treating cleft lip/palate, which may mean that these diagnoses are overrepresented in our dataset compared to the general population. Although the data may be influenced by skew, the aggregated data still provide insights into the distribution of procedures and their outcomes. Further studies could benefit from a more patient‐centered approach focusing on longitudinal data both within the same admission as well as during any readmissions. Further studies could also benefit from appropriate sampling strategies, to reduce the burden of data collection while accurately capturing volume and risk‐adjusted outcomes [45]. Finally, future work should adequately capture population level gaps in volume, access, and outcomes. This work should align with local and regional existing data collection strategies by clinical and public health leaders.

## 5 Conclusion

Assessing the epidemiology and outcomes of pediatric surgical care across 17 hospitals in 11 African countries provides important insights into the demographic, diagnostic, and procedural patterns of surgical care. It also evaluates progress made in the outcomes of pediatric surgeries in this setting and opportunities for improvement. Our analysis demonstrates the prevalence of surgery for conditions in neonatal patients, especially congenital malformations, and underscores higher mortality rates than in HICs especially in the youngest children. Overall, although this study provides a crucial snapshot of pediatric surgical care across diverse settings, it also serves as a call to action for policy‐makers, healthcare providers, and global health organizations to prioritize pediatric surgical infrastructure, training, and resource allocation. Addressing these challenges will be essential in reducing the mortality rates and improving the quality of surgical care for children across the continent. Future studies should aim to capture a broader spectrum of pediatric surgical needs, including those not yet addressed due to barriers in access, and explore more patient‐centered approaches to care, including longitudinal follow‐up and tracking of outcomes over time with lean and sustainable data collection approaches.

Ultimately, we hope this study will aid in advocating for investment in systems level capacity building, including prenatal care, referral pathways, and pediatric anesthesia, all of which are necessary for the quality delivery of surgical care for all children.

## Author Contributions

**Phillip J. Hsu:** formal analysis, investigation, methodology, project administration, software, visualization, writing – original draft, writing – review and editing. **Madeleine Carroll:** funding acquisition, investigation, methodology, visualization, writing – original draft, writing – review and editing. **Alan Zambeli‐Ljepovic:** formal analysis, investigation, writing – original draft, writing – review and editing. **Bolusefe Tijesuni Olatunji‐Banire:** formal analysis, investigation, writing – original draft, writing – review and editing. **Pawan Mathew:** formal analysis, investigation, writing – original draft, writing – review and editing. **Jason Axt:** data curation, investigation, resources, writing – review and editing. **Thierno Diallo:** data curation, investigation, resources, writing – review and editing. **Mekonen Eshete:** data curation, investigation, resources, writing – review and editing. **Bertille Ki:** data curation, investigation, resources, writing – review and editing. **Joseph Macharia:** data curation, investigation, resources, writing – review and editing. **George Ngock:** data curation, investigation, resources, writing – review and editing. **Absalat Serawit:** data curation, investigation, resources, writing – review and editing. **Emma Bryce:** data curation, investigation, resources, writing – review and editing. **Doruk Ozgediz:** data curation, funding acquisition, investigation, resources, supervision, validation, writing – review and editing. **Maija Cheung:** conceptualization, data curation, formal analysis, investigation, methodology, project administration, resources, supervision, validation, writing – original draft, writing – review and editing.

## Conflicts of Interest

The authors declare no conflicts of interest.

## Supporting information


**Table S1:** Top five conditions in the top three diagnosis categories.

## Data Availability

The data that support the findings of this study are available from the corresponding author upon reasonable request.
